# Torsion and the Homologous Ligation of Divided Arteries1Read before the section for Railway Surgery at the Pan-American Medical Congress, Washington, D. C., September 7, 1893.

**Published:** 1893-12

**Authors:** Thomas H. Manley

**Affiliations:** New York; Surgeon to the Harlem Hospital


Buffalo Medical and Surgical Journal

Vol. XXXIII. DECEMBER, 1893. No. 5.

### Original Communications.

### TORSION AND THE HOMOLOGOUS LIGATION OF DIVIDED ARTERIES.[1]

By THOMAS H. MANLEY, M. D., of New York.
Surgeon to the Harlem Hospital.

The question of prompt and effective suppression of hemorrhage is an all-important one to the surgeon, whose duties often bring him in contact with such serious railroad traumatisms as entail an extensive mutilation of the soft parts, with more or less destruction of the larger vascular conduits. Hence, as an elementary study, as it were, that concerning the subjugation of hemorrhage occupies a position of paramount importance to one dealing with this department of surgery. Therefore, both by study and practice, a full and useful knowledge of the principles which underlie hemostasis should be sedulously cultivated by us.

As our art is one without finality, he who would keep abreast of the times, and give his patient the benefit of the latest and the most effective therapy, must needs continue in a ceaseless search for that on which the least improvement can be made.

Recent advances in wound treatment, aseptic measures, absorbable ligatures, the elastic constrictor, and improved material for dressings, have all contributed to radically revolutionize views and methods which, until recently, had the support of dogmatic principles. Cellular-pathology, the studies of bio-chemistry, morphology, and histology, have struck a smiting blow at former doctrines on the definite occlusion of arteries. Animal experimentation has been an enormous aid in this line of investigation. It has been recently demonstrated that the rupture of the internal coat is not essential in the application of a ligature. (Ballance, on the

1. Read before the section for Railway Surgery at the Pan-American Medical Congress, Washington, D. C., September 7, 1893.

Ligation of Arteries in Continuity.) It has been proven, too, by the same author, that the clot is a foreign body in the vessel and does not undergo transformation into cellular tissue, as was formerly taught, but that the cellular infiltration is from the inner surface of the intima, which, though normally is permeated by no capillaries, in the event of injury it does possess the property of active cell-proliferation. It has been found that an absorbable, assimilable ligature is amply powerful to occlude the largest arterial trunks and may be safely imbedded in the tissues, when it is properly prepared, to subsequently soften, liquify, and be consumed by the phagocytes.

It has been further shown that simple approximation, without the division of the internal coat, is as effective in the application of a ligature to effect permanent cicatrization. Prof. Murdock and others have permanently dammed back the arterial current by the application of torsion, entirely dispensing with the ligature.

Esmarch's constructor cannot be denied an important place in the therapy of hemostasis, though all must admit that by its careless, injudicious, and too general application, it has worked great damage and has caused the needless sacrifice of many limbs and lives, which might have been spared without it.

But the possibility of its employment has taught us a useful lesson. It has demonstrated that the arteries may endure severe and protracted pressure without serious detriment to their integrity, and that the living current will long linger within the lumen of an artery without coagulating and plugging it.

But the severe pain which its pressure always entails is a warning to us that its general application is not without danger. Acupressure, acutorsion, and many other expedients have been alternately praised and condemned.

In a communication presented to the New York State Medical Association, in 1889 (*Transactions* of same year), I described what I designated the *Temporary Transfixion Ligature*, a simple and effective means by which any of the arteries of the extremities, large or small, could be promptly and safely controlled. Since then, Wyeth, of New York, and Senn, of Chicago, have each devised special methods to control hemorrhage in hip-joint amputations, both of which, in certain respects, combine the principles of this ligature; the former employing steel skewers, and the latter elastic tubing. At an early date I hope to again call the attention of the profession to this expedient.

Thus we see that many and various have been the measures employed to subdue arterial hemorrhage, and that recent advances in our knowledge of the *modus operandi* of the healing of arteries and prevention of sepsis permit us to apply measures heretofore quite inappropriate.

But the ideal is yet to be attained. The materials at our command even now are, in a large measure, unsatisfactory. All dead substances are unsatisfactory. Perhaps catgut is the most valuable when it can be secured fresh and is properly prepared, though often it is a source of infection. It may slip, stretch, or melt down before definite closure of the vessel is secured. Over the openings in small vessels, unless other tissues are included in the ligature, it is prone to slip off, so that it cannot be said to hold well, except on medium-sized vessels. Kangaroo, rat-tail, horsehair, and silk, are all objectionable, because of their irritating qualities.

Finding all heterogeneous materials more or less objectionable, and becoming better acquainted with the hemostatic properties inherent in the arteries themselves, surgeons have turned their attention to what might be accomplished by the utilization of the damaged segment of the vessel and the adjacent tissues as materials which might be utilized for the arrest of bleeding from arteries.

It had been observed that a very troublesome hemorrhage might follow the mere puncture of an artery, but that when it is cut cleanly across it immediately ceases. Vessels caught in the jaws of an artery-forceps, and held for a few moments, often promptly cease spouting when this is released. This, however, will not succeed in closing a vessel in continuity, unless the pressure is continued over considerable time, and a thrombus has formed at the proximal end. It has been often noticed that bleeding is seldom abundant from a large artery when a limb has been shot or torn off.

A wounded artery is essentially a structure seriously, if not totally, curtailed in function; blood circulating through it only under difficulties. All the tissues superjacent, or contiguous to it, become inflamed, and contract down on its walls; the vessel itself, after division, retracts within its sheath. The intima so coils on itself as to form a cuff of soft, filmy tissue, which, with the embolus, for the time at least, effectually aids in the arrest of hemorrhage. It has been claimed by Ballance, Zieler, Cornel, and Ranvier, that an embolus forms no essential part in the reparative processes of divided arteries.

My own observations incline me to the same view, for I wholly agree with those who regard the clot as always the result of septic infection, and, as Ballance so aptly puts it, "As much a foreign body in an artery as in the stump of an amputation." (Ballance and Edmunds, Ligation of Arteries in Continuity, page 127.)

There is every reason to believe, then, that auto-hemostasis is practicable when the secret methods by which it may be rendered efficient and practicable are revealed.

A long step in this direction was made when methodical asepsis became an accepted principle in wound treatment.

Baumgartner and Bottcher declare that no thrombus is ever formed if the vessel heals without suppuration (Ballance and Edmunds, pages 156 and 157). Perhaps, it is not sufficiently borne in mind that the various arteries of the body possess wide anatomical and physiological differences, and undergo certain definite changes at different stages in life, and that later researches, by more accurate and precise means than we now command, will show that there is a difference in the innate recuperative power, not only of various organs and parts, but in special elements of the same viscus, or member.

Dr. Murdock, after a careful survey of the history and principles of hemostasis, a long and ripe experience in general surgery, returned to first principles, with a strong conviction that with the aids which modern science have placed in our hands, the homologous occlusion of arteries as a general method in the hemostasis of traumatisms was feasible and practicable; therefore, he recommends the revival of the torsion of arteries on a large scale.

In Warren's late work on the Healing of Arteries, he tells us that Kocher, of Hamburg, related to Velpeau that he had not ligated an artery for twenty years; and that Velpeau himself was a strong advocate of torsion. The safety of torsion, he thought, depended on three things,—viz., the twisting of the external coat, retraction of the intima, and the coagulum. Ogston says (The Properties of Arteries and Their Ligation, p. 117,) that the normal blood pressure is from three to eight pounds to the square inch, and that a twisted vessel possessed less resistance than one ligatured.

I had but shortly cogitated seriously on the line of experimental work which had been confided to me by Dr. Murdock, when it occurred to me if the blood-current can be arrested through an artery by a twist of it, why not by a knot; in other words, why not tie it with its own elements, and therewith commit it to the grip of its own living walls, as well as occlude, by turning the vessel on its own axis?

With a determination of testing the practicability of torsion and homologous ligation, I commenced a series of experiments on the dog, and, as far as the opportunity offered, extended my observations on the human being, but, I regret to say, that since I commenced I have not had an opportunity to test this expedient on any of the larger vessels of man; and, because of many difficulties which will be removed later, I was unable to conduct my work with entire satisfaction on the dog.

In the present instance, my aim will have been accomplished if I succeed in simply calling attention to the possibilities in the future of securely occluding the bleeding orifices of arteries without the employment of any sort of foreign substance to embrace the artery; by torsion, when the cleavage of the vessel is on a line with the surface of the divided tissues, and by homologous ligation of the vessel, by means of its own walls, in those extensive mutilations in which, formerly, the surplus vessel was sacrificed with the other tissues. It had occurred to me whether or not, in many, we might not utilize a ribbon of the intermuscular, fibrous septa, the aponeurosis, or other fibrous tissues, for this purpose!

### OPERATIVE TECHNIQUE IN TORSION OF THE ARTERIES; IN EXPERIMENTAL PROCEDURES, AND ON THE HUMAN BEING.

Only the larger arteries were sought for.

The superficial femoral having been exposed and lifted out of its sheath, was seized between two clamps and divided with the scissors, directly across its long axis. There was an immediate separation of more than a centimeter of the ends.

The proximal end was put on extreme tension, when a second forceps was applied about two centimeters from the one which held the end. Now, the segment of the vessel was again stretched, when it was twisted until its outer superficial fibers were seen to rupture. Then, a fine suture was carried through the outer coat of the free end, to leave *in situ* for the purpose of drawing down the vessel in the event of torsion failing. The vessel was then liberated, when its twisted end disappeared in the tissues. There was no hemorrhage. The proximal end of the vessel having been safely disposed of, the distal was now taken up. Immediately on removing the clamp, which had held the orifice in a tight grip for ten minutes, blood spouted through it with great energy. The clamp was re-applied in the same position, with another a little more than a centimeter farther down, when the vessel was treated as the preceding, with the same result.

The common carotid on another dog was treated by torsion, which failed at the proximal end. I have tried this, experimentally, on no other arteries thus far; but, since taking up this study, I have generally employed nothing else as a hemostatic agent in occluding those smaller bleeding vessels, divided in abdominal and all surface operations.

The very few imperfect observations which I have made thus far on hemostasis by torsion, have convinced me that this is an expedient of great value in appropriate cases, when all its details of technique are fully carried out.

It would appear that it is especially valuable in those small vessels with thick coats of yellow elastic tissue. When these are imbedded in muscular tissue, torsion should wholly supplant every description of ligature. But there are many arteries which form channels for themselves in the tissues through which they pass, as those of the scalp, and others in which the vessels' coats and sheaths are so intimately incorporated with the parts through which they pass, as in the palmar surface of the hand and the plantar surface of the foot, that they do not permit of sufficient displacement forward and outward to allow of ample twisting for effective occlusion.

Enough has not been accomplished from experimental and clinical observations to enable us to estimate just how far torsion is a practicable and useful expedient in the obturation of the major arteries, though much has been written on the subject. A definite and precise manual is essential in the performance of torsion.

The bleeding orifice must be seized separately from other tissues. Gradual, but considerable, tension must be made on the free end of the vessel in order to clear it from adhesions with adjacent tissues, and, besides, to put the longitudinal fibers on a full stretch. Now, a second clamp seizes the vessel at its uppermost point, while a rotatory motion is commenced below, when the twisting is carried to the point of rupturing the outer coat. Then the forceps last applied is removed, when two or three more turns are given the end of the vessel. The latter is once more slightly stretched when we let go, and the stump of the vessel disappears in the tissues.

In order to give support to the mangled artery, and provide against secondary hemorrhage, it is always well to carry a light catgut suture through the tissues in which the crushed end of the artery is imbedded. In young people the collateral tributaries are so abundant that the distal end of the vessel must be effectually sealed before we finally close the wound, or complete the dressing. Now, the smooth, firm bandage over an abundance of aseptic, fluffy dressings, with the limb, stump, or part, in a comfortable position, the patient is placed on a proper regimen. Nature will promptly do the work of repair, and it may be said that in many cases the primary dressings need not be disturbed until the healing processes are complete.

This is the ideal to which we all should aim, but it is practicable and safe only under favorable circumstances. In order to realize it, every facility must be within easy command. Our patient, after operation, must always be placed in complete and permanent physiological rest until the healing processes are consummated.

### HOMOLOGOUS LIGATION.

Oftentimes, in the course of our studies, experimentation, and clinical observations, a new idea flashes through our mind, and at once, perchance, we imagine we have made a discovery.

There are today, remarkable to say, many who firmly believe that Harvey alone discovered the circulation, while admitting that centuries before this profound investigator was born the properties of the arteries and veins were well understood; and so there are others who would give all the credit of pulmonary physics to Lannaec, and, besides, proclaim that Lawson Tait was the first to discover purulent masses in the Fallopian tubes. But it is said that "There is nothing new under the sun," and, surely enough, soon after one has come forth and vaunted his new discovery, and feels himself safe as the sole recipient of its honors, great is his chagrin to learn that there is nothing original in his contribution, and that it had long since gone into oblivion because it possessed no practical value, and was hence cast aside as useless.

Is the same fate in store for the Homologous Ligation of the Arteries? Is there anything in the principle of utilizing the waste or excess of a vessel to close its own lumen?

Is there anything original in the utilization and appropriation of a living ligature to close a vessel?

Has this an application to other organic elements and structures besides the blood-vessels?

And, lastly, have others given this procedure a trial?

Does the theory rest on a substantial *rationale?*

Bone, we know, can only undergo repair or reproduction by bone. Destroyed integument can be replaced with the same tissue only. The same law applies to all the organic elements. Ballance tells us that even the most aseptic catgut, wound around an artery, is a foreign substance, which the phagocytes attack and assimilate as heterogeneous elements. All of which points, as this eminent author avows, to the fact that the ligation of arteries is yet in a very unsatisfactory state.

In the near past, when "laudable suppuration" was regarded as an important phenomenon in all flesh wounds, the presence of extraneous materials was not regarded as harmful. But, in our time, the elimination of suppuration, absolute and complete, is demanded as the *sine qua non* of sound surgery in all contaminated wounds. The methods of the past will not do for the present. Accordingly, in order to have our therapy in accord and harmony with modern biological studies and pathological doctrines, further advances must be made in many directions.

When the thought dawned on me that the homologous ligation of the arteries was a practicable expedient, it was soon apparent that it would only apply to certain definite phases of hemorrhage. This I was quite certain of, though it occurred to me that in many instances when the vital elements of the arterial walls were too scant to be used in this way, the heterogeneous tissues might be drawn on, in the immediate vicinity, for a similar purpose. Yet, there remained many situations in which obturation might be impracticable. In the vast majority of those cases, by the appropriate utilization of torsion, we can efficiently obturate the vessel.

Homologous ligation promises the most happy results in the larger arteries, as the femoral, brachial, carotid, and the larger arteries of the forearm and leg. It seemed to me a simple and practicable expedient in closing the arteries of the spermatic cord, as the cremasteric and spermatic are long, thread-like vessels, which could be firmly knotted when divided close to the gland.

This expedient seemed to me likewise specially adapted to those cases of primary amputation in which the limb is immediately and permanently destroyed beyond every hope, yet, in which many of the vessels and nerves preserve their vitality varying distances beyond the point at which an amputation must be made.

In the smaller arteries, when sufficient of the caliber of the vessel can be exposed without doing violence to adjacent parts, it might serve a most useful purpose.

That homologous ligation, in continuity, has a place in surgery, is yet to be determined. If, however, the principle on which it rests is a rational one, then there can be no question but that it may be utilized in this class with advantage.

### TECHNIQUE OF HOMOLOGOUS LIGATURE.

This may be divided into three stages :

1. *The isolation of the vessel.* 2. *Its bi-section and ligation.* 3. *Vascular fixation and approximation of the tissues.*

In the mangling of tissues, which we so generally find in very serious compound fractures, and when the trunk of the vessel to be treated lies free and close to the surface, under the torn integument and other tissues, it may be easily detached from its loose connections, raised from its bed, and drawn out sufficiently for homologous ligation without difficulty. But when the artery is torn in two, the question may arise as to whether, if the distal divided end preserve vitality, it may not, in this manner, be utilized for ligating the proximal end.

Assuming, however, that we propose dealing only with the proximal end and utilize it for its own occlusion, many difficulties may present themselves. If the brachial artery, as an instance, is torn through at the inferior end of the insertion of the pectoralis major, close to where the internal and external cords of the brachial plexus form a Y, and the main trunk passes immediately into and under it, the isolation of it without damage to the nerves, or sacrifice of many of the numerous axillary branches, may seem quite impossible.

In an amputation we may have little consideration for those contingencies. Hence, if a large nerve trunk lies in the way, we divide it, lift the vessel up through the gap, then replace the nerve ends, pass a fine catgut suture through the neurilemma, and replace the nerve. If the section of the main trunk is convenient to a large branch, in amputation cases, this may be caught between two forceps and divided.

At least two inches of the extent of the vessel must be liberated and exposed with its sheath intact if possible, when gradual but steady tension is made on the vessel in the direction of the long axis of the trunk until it is fully stretched; at which time another clamp forceps is firmly fixed as close to its point of emergence from the tissues as possible. This completes the first stage of the operation.

### BI-SECTION AND LIGATION.

It will be noticed that thus far our initiation varies but slightly from that of torsion, which, indeed, this is a species of. Its contrasting features consist only in that instead of twisting the vessel on itself we utilize its walls for a knot.

The clamp on the free end of the vessel is now removed, and we will deal with it according to its caliber in two different ways. If it be a smaller vessel, it is tightly knotted on itself in two or three places. When each knot is drawn, tension is maintained until the elasticity of the vessel is overcome; and now that the knotting is completed, the vessel is liberated.

With a large trunk, a somewhat different technique must be observed. The initial steps, however, are similar to those employed on more diminutive vessels. The vessel is bisected or trisected, while I have succeeded by merely dividing the vessel and tying either end. Later experiments have assured me that to split the vessel in three segments, employing one to turn on itself and plug the open lumen of it, and then ligate with the other, a more effective obturation is secured than on using but two strands. In all these cases of section of the vessel, the division was carried up to within about two lines of the point, at which the uppermost margin of the artery appeared through the tissues. Now the free ends are again moderately stretched, when they are tied with a reef-knot, singly, doubly, or trebly. The knotting being completed, the free ends are firmly drawn on and tension continued from two to four minutes, when the upper forceps are removed and the vessel is released.

The third and final stage embraces *Vascular Fixation*, and approximation of the tissues. With the larger vessels, with this procedure, an adjustment ligature of strong catgut, carried well into the tissues and firmly knotted, serves a most valuable auxiliary purpose. The integumental suture will serve this purpose in the majority of cases, but if an accessory suture or two are regarded as necessary, we should not hesitate to insert them.

It goes without saying that in every instance the possible success of homologous ligation of the arteries depends on an aseptic wound.

Every description of straining and bodily movement should be prevented, as far as possible, for the first forty-eight hours after operation. Vomiting, coughing, or straining at stool, all greatly increase arterial tension, and should be avoided until the immediate danger of secondary hemorrhage is past.

### CONCLUSIONS.

Whether homologous ligation or obturation of arteries, the former by a living tissue, and the latter by torsion of the vessel, possess any enduring value, experimental work and clinical observations alone will decide. Torsion is a very ancient expedient, which suffices for the diminutive and superficial vessels; but, for those very deeply lodged, or those of very large caliber, it has not been very generally trusted until recent times. Since the attention of the profession has been called to the subject by Prof. John B. Murdock, who has revived it on a large scale, and has shown that with the perfected technique, with modern dressings, and the elimination of suppuration from fresh wounds, its application has a very large field.

As for homologous ligation, I am firmly convinced that, with the onward march of progress, in the near future, the question of perfected hemostasis will be definitely settled, and that on the experimental line of investigation its solution will, in the main, rest.

Time and opportunity permitting, it will be my aim to continue my experimental work, and test to the fullest this species of automatic obturation in the human subject, and report final results later.

### EXPERIMENTS ON THE ARTERIES OF THE DOG—TORSION TRANSFIXION LIGATURE, AND HOMOLOGOUS LIGATION.

June 15, 1893, middle-sized terrier poodle. Animal anesthetized with ether. Right femoral artery exposed in Scarpa's triangle for about half an inch of its extent. Its sheath was divided, the wall of the vessel lifted out, when it was caught, and the entire caliber of the trunk compressed by two coarsely-toothed, strong, clamp forceps. These were allowed to remain on ten minutes, when they were removed. Immediately the lumen of the vessel refilled, and the pulsations were as vigorous as ever beyond the point of compression.

Now the two forceps were again applied over their previous site, when the vessel was divided with the scissors completely through, after which the distal forceps was seized, and slight but steady traction made for a moment, when it was made to twist the open end of the vessel until that extent of torsion was reached that it could be carried no further without effecting the complete detachment of that part of the vessel engaged. Both forceps, at this stage, were removed. The free orifice of the vessel had been completely occluded. It freely pulsated with each systole of the heart, but hemostasis had been complete. After the divided parts had been duly prepared, they were approximated and covered with dressings.

### ACCIDENTAL DIVISION OF THE LEFT FEMORAL ARTERY—PROMPT AND EFFECTIVE HEMOSTASIS BY THE TEMPORARY TRANSFIXION LIGATURE.

It was intended to repeat the same experiment on the left femoral, at the same time, on the same animal. Not being aided by ample assistants, and finding that the vessels on this side pursued a rather abnormal course, the artery was accidentally wounded and immediately gave issue to large jets of blood. As it had been completely cut in two, both ends were quickly retracted out of sight in the soft parts.

In this emergency, as ample facilities were not at hand to search for and secure the spouting proximal end, the trunk was compressed over the pubis, when a silk ligature, with a sharply curved needle, was passed under the proximal segment of the vessel, about half an inch posterior to the orifice. This ligature included the loose connective tissue, the sheath, *vasa vasorem*, artery, and other structures. After being tied, one free end was left out. Thus, the transfixion ligature effectually succeeded in closing the lumen of the vessel and subduing all hemorrhage. The incised parts were closed in by one row of continued suture, when dressings were applied.

The dog came out of ether well, and was returned to his crib June 30th.

The wounds over the inner side of the thighs were at this time nearly healed. It was now intended to extend our investigations into the carotid triangles by torsion on one vessel and homologous ligation of the artery on the other side.

The animal was placed under ether, and the sheath of the vessel exposed on the right side, when it was noticed that he ceased to breathe. In a moment he was dead.

Now, a very careful examination was made over the site of the incisions, which exposed the femoral vessels, two weeks previously. The right side was the first dealt with. Here it was found that all the tissues had cicatrized into one homogeneous mass.

Tearing open this cicatrix, no trace of the artery could be found until about two centimeters of the scar tissue was stripped in the direction of the long axis of the limb in an upward direction, at which point the obturated cord-like proximal end of the artery was discovered, though it was so intimately incorporated with the cicatrix, in which it was imbedded, as to be only separated with difficulty. By tracing this up a few lines, we came on the artery, which was empty, except for a line above the point of obliteration, at which point a decolorized, conical blood-clot was found. The embolus was, even at this early stage, so blanched, consolidated, and organized, as to clearly resemble connective tissue.

The distal end was not so deeply decolorized as the proximal. Its lumen was obliterated, not by an embolus, but by an adhesion of its walls from an exudative endarteritis.

### DISSECTION OF THE LEFT FEMORAL ARTERY.

On this side the *temporary transfixion ligature* had been employed. Although the purpose of this ligature is to only serve a momentary end in this case, it was allowed to remain on for two days, after which time it was cut away, when no hemorrhage followed.

In this dissection, as the former, it was seen that the injured artery was caught and entangled in a mass of new tissue.

But it was continuous, though, from a point immediately below Poupart's ligament, but the trunk of the femoral artery was obliterated for about five centimeters, when the open lumen below was reached.

As with the vessel on the right side, the proximal end was firmly occluded by an organized, firmly adherent embolus.

It may be added that in both of these extremities, in which simultaneously the femoral torrent was abruptly cut of, they remained well nourished, and, on *post-mortem* examination, there were no evidences of atrophic, wasting changes.

EXPERIMENTS ON SECOND ANIMAL.

July 6, 1893. (1) Homologous ligation of the proximal end of the right carotid artery in the superior carotid triangle. Closure of the distal end by torsion. Completely successful. (2) Homologous ligation of the right femoral artery in Scarpa's triangle. As the knot slipped, after the proximal end retracted, the hemorrhage was now controlled by the temporary transfixion ligature. The distal end of the artery was closed by torsion.

July 17th. Same animal, a bull-terrier, examined. All the ligatures held. There was a moderate-sized exudation into the subcutaneous tissues at the point where the carotid was divided; neither of the wounds healed; primary union. Again anesthetized and homologous ligation of the left femoral artery undertaken. Here, on division of the tissues, such an anomalous state of things was discovered as to render this species of arterial obturation rather impracticable. The vessel bifurcated in Scarpa's triangle, and so wound around the femoral vein as to render its isolation very difficult. Besides, the vein itself was lacerated, and gave issue to a large effusion of blood before it could be controlled. In this case, as the artery was wounded on manipulation, and it was found necessary to apply a ligature directly over its proximal and distal ends, our experiment on this occasion was an entire failure.

It was noticed, so far, in our experimental work, that the collateral branches of the arteries in the dog are large and numerous, and, hence, the suppressed circulation is very promptly reëstablished. This is apparent by the energy of the recurrent torrent from the distal end of the vessel after division; indeed, complete obturation is as necessary here as with the cardiac end of the vessel.

A singular peculiarity was noticed with the veins of the dog. Their walls are proportionally very much thicker than with man, and the energetic circulation through them demand well-directed pressure to arrest it.

MISCELLANEOUS APPLICATION OF THE PRINCIPLE OF HOMOLOGOUS OBTURATION IN CLOSING THE LUMINA OF CAVITIES AND SPACES.

Since this line of study and experimentation has occupied my attention, it has occurred to me that if homologous obturation of the arteries is feasible and practicable, then the field for the general utilization of the living ligature, or suture, was, indeed, a large one.

On the 20th of July, this Summer (1893), a captain in the fire department of this city came into my hospital service with a strangulated hernia. On decortication of the sac, under cocanization, with a redundant flap of fibro-serous tissue, in my hand, the thought occurred to me, why not utilize this tissue to obturate the inguinal canal? Authors speak of stretching, twisting, furling, ligating, and excising the sac, in hernial operations; but none, that I am familiar with, have yet recommended utilizing it as a knot for tying into a knot and closing in the ring.

In this instance, I split the sac in the direction of its long axis, carrying the incision as far upward as the internal ring. I then seized two loops and tied them in a triple-reef knot. After the knot was fixed, it was pressed inward within the internal ring. Then the other two loops were so tied as to overlap the first knot. This was secured in a double-reef knot, when a catgut suture was carried on a needle directly through it and the edges of the pillars on either side, then firmly tied.

The incision was closed without drainage. Healing was prompt; constitutional symptoms, *nil*; and our patient left the hospital, soundly healed, in just two weeks.

Two weeks ago I resected a large plexus of veins on the inner aspect of the leg and thigh of a young woman, when I found that the homologous ligation of either ends of the vessels served an admirable purpose as a ready and effective means of obturation. The vessels were greatly dilated, and quite valueless, so that the recurrent flow from the proximal divided end of the saphenous required firm pressure to close it. But the living suture was equal to the occasion.

Cases might be multiplied in which this expedient serves an ideal purpose. When there is a sparcity of homologous tissue, there is no reason why we should not reach out and utilize or borrow from a neighboring structure, as from the muscular sheaths, aponeuroses, the nerve sheaths of nerves no longer needed, tendon, integment, etc.

Without doubt, other investigators and operators will so perfect and extend the employment of this principle that, in the near future, the technique will be so simplified and effective that no sort of extraneous ligature, or suture material, will be required for any only very exceptional cases. Man, undoubtedly, carries within himself all the necessary materials for the mechanical repair of many traumatic lesions to which he is liable.

Time has proven that all chemical solutions are foreign substances, and that they have no place in healthy wounds; that the ideal is asepsis, sterilization, and non-irritant liquids; that over-draining and excessive flushing are both needless and harmful. May not the time be near when the question of ligation of arteries and approximation of tissue by foreign materials will be solved by discarding generally all except such as are provided by the living economy?

The successful utilization of the autogenous ligature, or suture, depends chiefly on rigorous asepsis. It has no place in a foul infected wound. Those operations in which it is employed are necessarily rendered more tedious, as effective technique entails very delicate manipulation. In all cases of homologous ligation of the blood-vessels, one should be assured that all hemorrhage is subdued before the wound is finally sealed. The parts must be extremely quiescent for the first twenty-four hours. After that, with ordinary care, everything will go on well, and, as a rule, not more than one dressing is necessary until union is complete.

